# Functional evaluation of complement factor I variants by immunoassays and SDS-PAGE

**DOI:** 10.3389/fimmu.2023.1279612

**Published:** 2023-10-26

**Authors:** Alexandra Gerogianni, Laura M. Baas, Dick J. Sjöström, Nicole C. A. J. van de Kar, Marit Pullen, Siem J. van de Peppel, Per H. Nilsson, Lambertus P. van den Heuvel

**Affiliations:** ^1^ Linnaeus Centre for Biomaterials Chemistry, Linnaeus University, Kalmar, Sweden; ^2^ Department of Chemistry and Biomedicine, Linnaeus University, Kalmar, Sweden; ^3^ Department of Pediatric Nephrology, Radboud University Medical Center, Amalia Children’s Hospital, Nijmegen, Netherlands; ^4^ Department of Genetics, Radboud University Medical Center, Nijmegen, Netherlands; ^5^ Department of Pediatrics/Pediatric Nephrology, University Hospitals Leuven, Leuven, Belgium; ^6^ Department of Development and Regeneration, University Hospitals Leuven, Leuven, Belgium

**Keywords:** factor I, co-factor activity, functional assay, complement regulation, factor H, complement receptor I

## Abstract

Factor I (FI) is an essential regulator of the complement system. Together with co-factors, FI degrades C3b, which inhibits further complement activation. Genetic mutations in FI are associated with pathological conditions like age-related macular degeneration and atypical hemolytic uremic syndome. Here, we evaluated eight recombinant FI genetic variants found in patients. We assessed FI’s co-factor activity in the presence of two co-factors; Factor H and soluble CR1. Different analytical assays were employed; SDS-PAGE to evaluate the degradation of C3b, ELISA to measure the generation of fluid phase iC3b and the degradation of surface-bound C3b using a novel Luminex bead-based assay. We demonstrate that mutations in the FIMAC and SP domains of FI led to significantly reduced protease activity, whereas the two analyzed mutations in the LDLRA2 domain did not result in any profound changes in FI’s function. The different assays employed displayed a strong positive correlation, but differences in the activity of the genetic variants Ile55Phe and Gly261Asp could only be observed by combining different methods and co-factors for evaluating FI activity. In conclusion, our results provide a new perspective regarding available diagnostic tools for assessing the impact of mutations in FI.

## Introduction

The complement system is an essential part of innate immunity and consists of around 50 plasma- and membrane proteins for host surveillance, including recognition and elimination of pathogens and cell debris (1). Complement activation can arise through three different routes; the classical, lectin, and the alternative pathways. All pathways result in the proteolytical activation of zymogens, including cleavage of the central complement components C3 to C3a and C3b, and C5 to C5a and C5b, which leads to effector functions, including C3b-opsonization, generation of C3a- and C5a-anaphylatoxins and formation of the terminal C5b-9 complement pathway (2). The alternative pathway, activated by surface-deposited C3b or the fluid phase analog C3(H_2_O), acts as an intrinsic amplification loop for all pathways. Therefore, tight regulation of the alternative pathway on host cells is critical, as insufficient inhibition of C3b can lead to uncontrolled complement activation and excessive inflammation (3, 4).

Factor I (FI) is a key negative regulator of complement activation. In the presence of its co-factors, factor H (FH), factor H-like protein 1 (FHL-1), complement receptor 1 (CR1; CD35), and membrane co-factor protein (MCP; CD46), FI can cleave the α´-chain of C3b into iC3b, and in the presence of CR1, FI can further degrade iC3b into C3dg and C3c (2, 5–7). FI circulates in a zymogen-like state until the C3b/co-factor complex is engaged (8, 9). Then, FI is conformationally changed and able to cleave C3b (10). FI is a heterodimer with a molecular weight of 88 kDa (9). The non-catalytic heavy chain consists of a FI membrane attack complex (FIMAC) domain; a scavenger receptor cysteine-rich (SRCR) domain; two class A low-density lipoprotein receptor domains (LDLRA1 and LDLRA2); and a C-terminal region of unknown function, while the catalytic light chain has a chymotrypsin-like serine protease (SP) domain (7, 8).

Mutations in FI can impair its secretion or enzymatic function, which can lead to tissue damage and disease (11–13), including age-related macular degeneration (AMD) and atypical uremic syndrome (aHUS) (9, 10, 14). In both diseases, insufficient complement regulation drives the disease pathogenesis (15, 16).

Carriers of rare *CFI* variants were reported with younger age of AMD symptom onset (17), and loss of function FI variants are reported to have the greatest impact on the risk of developing the disease (18). It was demonstrated that rare variants in the SP domain of the *CFI* gene were associated with AMD (19). Furthermore, mutations in the FIMAC domain seem to interfere with FI-mediated C3b degradation (20). Mutations in the *CFI* gene are found in a relatively low incidence (<5%) in aHUS patients. However, the risk of recurrence was higher after kidney transplantation in carriers of pathogenic *CFI* variants (21–23).

The functional consequence for the majority of the *CFI* gene variants has not been examined yet, highlighting the need for functional characterization of genetic variants. Next to this, functional characterization of *CFI* genetic variants is done differently across laboratories, and standardization is lacking. The co-factor activity of FI may occur on either surface-bound C3b, or C3b in the fluid phase and to date, no comparisons between surface-bound and fluid-phase C3b degradation by FI have been reported.

To further investigate the pathophysiological consequences of carriers of rare *CFI* variants, we evaluated the functionality of eight recombinantly expressed FI-variants by both surface-bound and fluid phase C3b using three different methods (SDS-PAGE, ELISA and Luminex technology) with both FH and sCR1 as co-factor. Finally, we evaluated the correlation of these three functional readouts for FI activity and the usage of the two different co-factors.

## Materials and methods

### Reagents

Human complement proteins FH and FI were bought from CompTech (Tyler, TX). Human C3 was purified as described by Hammer et al. (24). Human C3b was produced by trypsin digestion of C3. C3 was incubated for five minutes at 22°C with 1% (w/w) trypsin from bovine pancreas (Merck; Kenilworth, NJ) and isolated with gel filtration on Sephadex G100 (GE Healthcare, Chicago, IL) followed by equilibration with 10 mM phosphate buffer, pH 7.4, and 0.145 M saline (PBS). C3b was also purchased from CompTech. Soluble CR1 (sCR1) was obtained from T Cell Sciences Inc. (Cambridge, MA).

### *CFI* mutants

**Figure 1:**
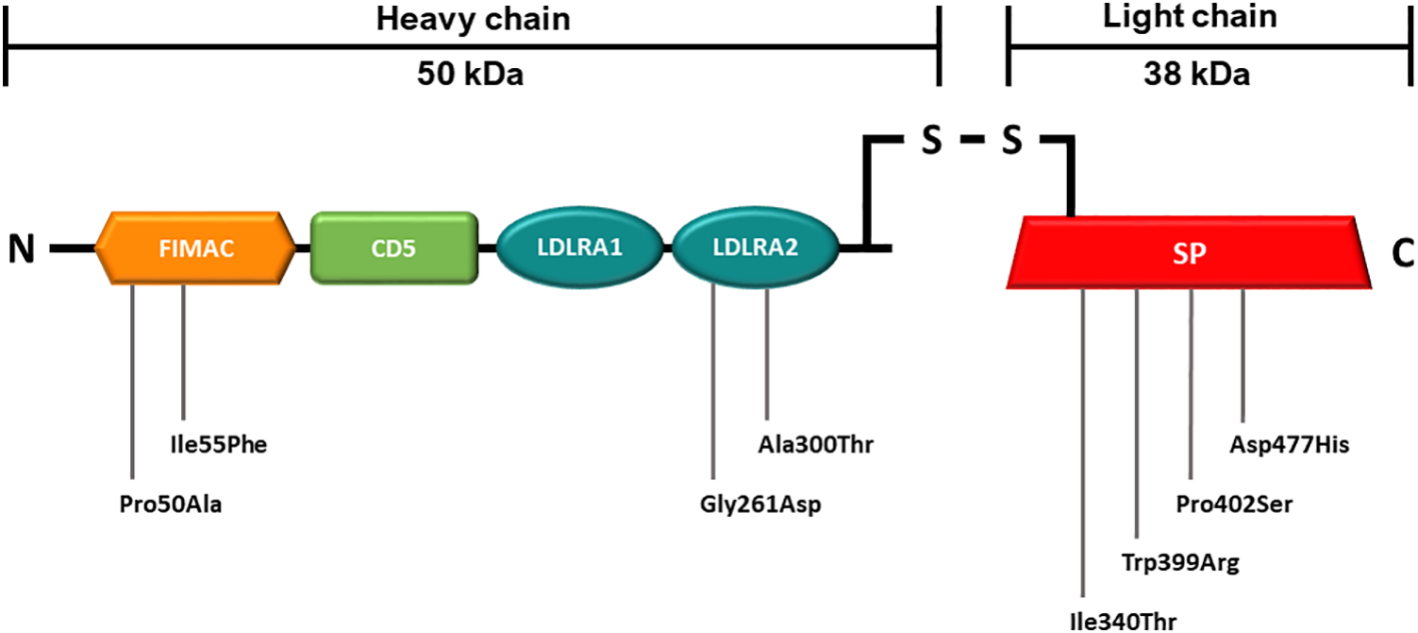
Schematic representation of the mature FI protein with mutations analyzed in this study. The 50 kDa heavy chain consists of four domains which are are shown in orange (FIMAC), green (CD5) and blue (LDRA1 and LDRA2). The 38 kDa light chain consists of one domain which is shown in red (SP). Details on the genetic variants are summarized in **Table 1**. FIMAC, Factor I-Membrane Attack Complex; CD5, scavenger receptor cysteine-rich domain; LDLRA1 and LDLRA2, Low density lipoprotein receptor domains 1 and 2; SP, Serine Protease domain.

The *CFI* mutations were identified in the cohorts of aHUS and AMD patients at the Radboudumc Genetics department (Nijmegen, Netherlands). *CFI* variants are presented in **Table 1** and their distribution throughout FI is shown in **Figure 1**.

**Table 1:** Overview of FI genetic variants and recombinant proteins.

cDNA change	Protein change	rs-number	Domain	GenomAD frequency (ALL)	Classification	Clinical phenotype
**148C>G**	Pro50Ala	rs144082872	FIMAC	0.01%	3	AMD and aHUS
**163A>T**	Ile55Phe	N/A	FIMAC	–	3	AMD
**782G>A**	Gly261Asp	rs112534524	LDLRA2	0.13%	2	AMD and aHUS
**898G>A**	Ala300Thr	rs11098044	LDLRA2	1.1%	1	AMD
**1019T>C**	Ile340Thr	rs769419740	SP	0.0073%	4	AMD and aHUS
**1195T>C**	Trp399Arg	rs778871974	SP	0.002%	2	AMD and aHUS
**1204C>T**	Pro402Ser	rs1416762682	SP	–	3	aHUS
**1429G>C**	Asp477His	rs754972981	SP	0.002%	3	AMD

Classification of the variants was done according to previously described guidelines (25) as benign (Class 1), likely benign (Class 2), uncertain significance (Class 3), likely pathogenic (Class 4) and pathogenic (Class 5). cDNA and protein changes were determined using transcript NM_000204.4.

### Generation of recombinant FI

*In vitro* mutagenesis was performed on a pcDNA3 vector (ThermoFisher; Waltham, MA) containing the full-length wild-type *CFI* cDNA (ACCN NM_000204.4; GRCh37) using the Q5® Site-Directed Mutagenesis Kit (New England Biolabs; Ipswich, MA) with specific primers to introduce each variant into the wild-type gene (for primer sequences see **Supplementary Methods, Table 1**). After PCR, samples were digested with DpnI (ThermoFisher) for one hour at 37°C. Obtained plasmids were transformed into the *E.coli* DH5-α strain with heat pulse, after which the bacteria were selected on LB plates containing 50 µg/mL carbenicillin. DNA samples were purified using the PureYield^™^ Plasmid miniprep and midiprep kit (Promega; Madison, WI) according to the manufacturer’s instructions. Fidelity of the clones was confirmed by PacBio sequencing. For the expression of recombinant protein, Human Embryonic Kidney 293T (HEK293T) cells were maintained in DMEM (ThermoFisher) supplemented with 10% FCS (ThermoFisher), 2 mM L-glutamine (ThermoFisher), 1% MEM non-essential amino acids solution (ThermoFisher), 1 nM sodium pyruvate (Merck), and 100 units/mL penicillin-streptomycin (ThermoFisher) and were seeded onto culture vessels pre-coated with 20 µg/mL collagen I (Merck). When the HEK293T cells reached 70-90% confluency, transfection was performed using lipofectamine 2000 transfection reagent (ThermoFisher). Forty hours post-transfection, the cells were washed twice with PBS, after which Opti-MEM (ThermoFisher) was added to the culture vessel. Supernatants were collected 55 hours post-transfection and subsequently concentrated with Amicon® Ultra 10kDa (Merck) following manufacturers’ instructions.

### Evaluation of FI recombinant proteins

*Protein concentration:* FI protein concentration in the concentrated supernatants was determined by ELISA as described previously (12, 26). In summary, a 96-wells high-binding plate (Greiner; Kremsmünster, Austria) was coated with a sheep anti-human FI antibody (LabNed; Edison, NJ) diluted 1:1000 in carbonate buffer pH 9.6 (15 mM Na_2_CO_3_·10H_2_O, 34.5mM NaHCO_3_) and incubated overnight at 4°C. The plate was blocked for 30 minutes with superblock (ThermoFisher) and subsequently with PBS supplemented with 1% bovine serum albumin (BSA) for 1 hour at room temperature (RT). Following incubation, the plate was washed with PBS 0.05% v/v Tween-20 after blocking and between each of the following steps. Samples and the standard (serum-purified FI, CompTech) were diluted in PBS containing 0.05% v/v Tween-20 and 0.2% BSA and afterwards incubated for two hours at RT. After applying the samples, FI was detected with a mouse anti-human FI antibody (OX-21, ProSci; Fort Collins, CO) diluted 1:1000 in PBST-0.2% BSA and incubated for two hours at RT. After incubation, the secondary goat anti-mouse HRP antibody (Dako; Glostrup, Denmark) was diluted 1:2500 in PBST-0.2% BSA and incubated for 1 hour at RT. Detection was executed using o-phenylenediamine dihydrochloride substrate (OPD; Merck) diluted according to the manufacturer’s instructions and incubated for 30 minutes at RT after which the reaction was stopped with the addition of 1 M H_2_SO_4_. Absorbance was measured at 492 nm by Tecan Spark 10M multi-mode microplate reader (Männedorf, Switzerland). All samples were measured in duplicates.

*Protein identification:* The recombinant proteins were diluted in Laemmli sample buffer enriched with 1:20 β-mercaptoethanol and incubated for 5 minutes at 95°C. Samples were separated on a 4-15% Mini-PROTEAN® TGX™ gel (Bio-Rad; Hercules, CA) for one hour at 120 V. Separated proteins were transferred to a 0.45 µm polyvinylidene fluoride (PVDF) membrane (Bio-Rad) for 7 minutes using the Trans-Blot® Turbo™ Transfer System (Bio-Rad), after which the membrane was blocked with PBS containing 5% milk for one hour at RT. The membrane was incubated overnight at 4°C with a goat anti-human FI antibody (Quidel, San Diego, CA) diluted 1:500 in PBS containing 5% milk. The membrane was washed three times for 5 minutes with PBS 0.05% Tween-20 on a rocking platform and incubated with the secondary donkey anti-goat HRP antibody (Dako) diluted 1:2000 in PBST containing 5% milk. After incubation, the membrane was washed three times for 5 minutes with PBS 0.05% Tween-20, and chemiluminescence was detected using the SuperSignal™ West Femto Maximum Sensitivity Substrate (Thermo Fischer).

### SDS-PAGE

C3b (10 μg) was mixed with recombinant FI (0.1 μg) together with its co-factors (0.1 μg) FH or sCR1 and incubated in PBS for 60 minutes. Samples that contained C3b, FH, or sCR1 with and without FI (CompTech) were used as positive and negative controls, respectively. The total reaction volume was 10 μL, and the incubations were performed in 1.8 mL Nunc-cryotubes (Nunc; Roskilde, Denmark) at 37°C. After incubation, 5x SDS-PAGE reduced sample buffer (Bio-Rad) was added, and the samples were heated for 10 minutes at 95°C. The samples were applied on 12-wells 4 – 15% Tris-glycine gels and applied to SDS-PAGE with a molecular marker (Bio-Rad). The gels were stained with SYPRO Ruby protein stain (Bio-Rad) and evaluated using Chemi Doc Imaging System (Bio-Rad).

### iC3b ELISA

FI recombinant proteins and the plasma purified FI positive control (CompTech) were diluted to 2 µg/mL in Tris-buffered saline pH 7.4 (TBS, 50 mM TrisHCl, 150 mM NaCl). C3b (15 µg/mL) and FH (3 µg/mL) were added to each sample and the reaction mixture was incubated for three hours at 37°C. C3b degradation was analyzed by detecting iC3b concentrations with ELISA. A high-binding microtiter plate (Greiner) was coated with mouse anti-iC3b (Quidel), diluted 1:2500 in a 50 mM carbonate buffer pH 9,6), by overnight incubation at 4°C and subsequently blocked with PBS 1% BSA for one hour at RT. The plate was washed with PBS 0.05% Tween-20 (PBST) after coating, blocking and between each of the following steps. Samples were diluted in PBST and incubated for 1 hour at RT. iC3b was detected with a rabbit anti-human C3c (Siemens; Erlangen, Germany) diluted 1:2000 in PBST with 0.2% BSA and incubated for 1 hour at RT followed by the secondary goat anti-rabbit HRP antibody (Dako) diluted 1:2000 in PBST with 0.2% BSA and incubated for one hour at RT. O-phenylenediamine dihydrochloride (OPD) (Sigma) was added as a substrate for the HRP and incubated for 30 minutes at RT after which the reaction was stopped with the addition of 1 M H_2_SO_4_. All samples were measured in duplicates. Absorbance was measured at 492 nm by Tecan Spark 10M multi-mode microplate reader (Männedorf, Switzerland).

### Luminex assay

*Conjugation of magnetic beads with C3b:* C3b was conjugated to magnetic beads as described previously (27). Briefly, cystamine sulfate hydrate (6 μg, Merck) was coupled to Bio-Plex Pro™ magnetic COOH beads (Bio-Rad) with the use of the amine coupling kit (Bio-Rad) following the manufacturer’s instructions. The beads were then incubated with dithiothreitol (DTT; Roche Diagnostics, Rotkreuz, Switzerland) at 6 μM final concentration for 20 minutes at 22°C to reduce the internal disulfide bond of cystamine. After thorough washing with 10 mM phosphate buffer and 0.145 M saline (PBS) at pH 7.4, C3 (15 μg) and trypsin (1 μg, Merck) were added to the beads and were incubated for 10 minutes at 37°C to induce C3 degradation resulting in C3b linkage to the beads via the thioester. After the incubation, the beads were washed thoroughly with PBS to end the reaction.

*Assay:* C3b magnetic beads were added to a black flat-bottom 96-well plate (Bio-Rad) containing 3000 beads/well with PBS with 0.1% Tween 20 and 0.05% BSA. After aspiration, recombinant FI (0.4 μg) and sCR1 (0.4 μg) were added to the wells to a total volume of 50 μL. C3b beads incubated with trypsin (140 μg/mL) and PBS were used as positive and negative control, respectively. All samples were incubated for one hour at 37°C on agitation (800 rpm). The reaction was stopped by washing three times with PBS 0.05% Tween-20. C3b degradation was evaluated by detecting the loss of the C3c-signal derived from bead-bound C3b and iC3b by using a biotinylated polyclonal rabbit anti-human C3c antibody (4 μg/mL, Dako) followed by washing and addition of streptavidin-PE (1:100, Bio-Rad). In both steps, the microplate was incubated for 30 minutes at room temperature. All reagents were diluted with PBS with 0.1% Tween 20 and 0.05% BSA. Mean fluorescent intensity (MFI) was determined using Bio-Plex MAGPIX Multiplex reader.

### Data analysis and statistics

The SDS-PAGE data were analyzed by Image J (US National Institutes of Health, https://imagej.nih.gov/ij). Data visualization, absorbance spectroscopy calculations, and statistical analysis were performed using GraphPad Prism version 8.0.1 for Windows or MAC (San Diego, CA). Comparisons between multiple columns were analyzed with the one-way ANOVA with Dunnett’s multiple comparison test. Pearson correlation coefficient was computed for the comparison of two variables.

### Ethics statement

This study met the criteria of the Declaration of Helsinki (28).

## Results

### Degradation of fluid phase C3b (SDS-PAGE)

**Figure 2:**
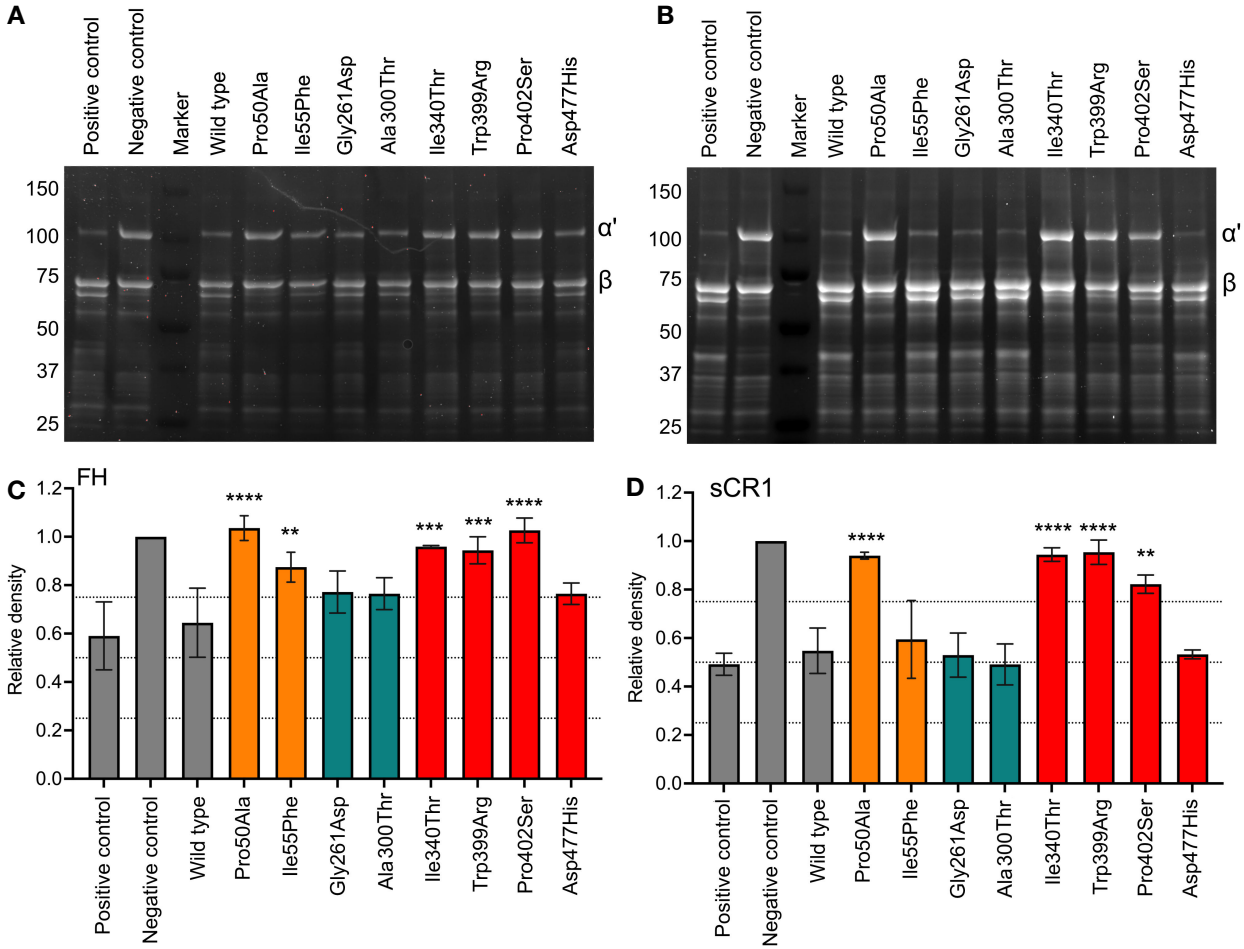
Functional analysis of recombinant FI proteins with SDS-PAGE. FI recombinant proteins (0.1 μg) were incubated with C3b (10 μg) and **(A, C)** FH (0.1 μg) or **(B, D)** sCR1 (0.1 μg) for 60 minutes at 37°C. The samples were reduced and applied onto a 12-well 4-15% SDS-PAGE. The gels were stained with SYPRO Ruby protein stain. Gels displayed **(A, B)** are representative of three independent experiments. Degradation of C3b was measured by densitometry and shown as the relative density of the intact C3b α´-chain (101 kDa) in relation to the β-chain (75 kDa). Positive and negative control samples, and wild-type (WT) are indicated in grey, FI protein domains are color coded according to: FIMAC domain in orange, LDLRA2 domain in blue and SP domain in red. The values are shown as mean +/- standard deviation of n=3. **p <0.01, ***p <0.001, and **** p <0.0001.

SDS-PAGE was applied to evaluate FI-mediated cleavage of the C3b α´-chain in the fluid phase in the presence of co-factors FH (**Figures 2A, C**) and sCR1 (**Figures 2B, D**). The eight recombinantly expressed FI-variants and the recombinantly expressed wild-type FI (**Supplementary Figure 1**) were evaluated in parallel to FI purified from human plasma. With FH as a co-factor, most FI mutants showed attenuated C3b degradation in comparison to both the recombinantly expressed wild type and the FI purified from plasma (**Figures 2A, C**). Mutations in the FIMAC domain, Pro50Ala and Ile55Phe, resulted in significantly less C3b α´-chain cleavage (p<0.0001 and p<0.01, respectively) compared to the WT. All of the mutations of the SP domain, apart from Asp477His, resulted in reduced C3b-degradation when compared to the WT; Ile340Thr and Trp399Arg (both p<0.001), and Pro402Ser (p<0.0001). No significant difference was observed for the analyzed mutations in the LDLRA2 domain. The degradation pattern of C3b with sCR1 was similar to that of FH (**Figures 2B, D**). Pro50Ala, Ile340Thr, Trp399Arg and Pro402Ser showed significantly less C3b-degradation than the control (p<0.05-p<0.0001). However, the two mutants in the LDLRA2 domain, Asp477His in the SP domain and Ile55Phe of the SP domain were similar to the WT and positive control.

### Generation of iC3b (ELISA)

**Figure 3:**
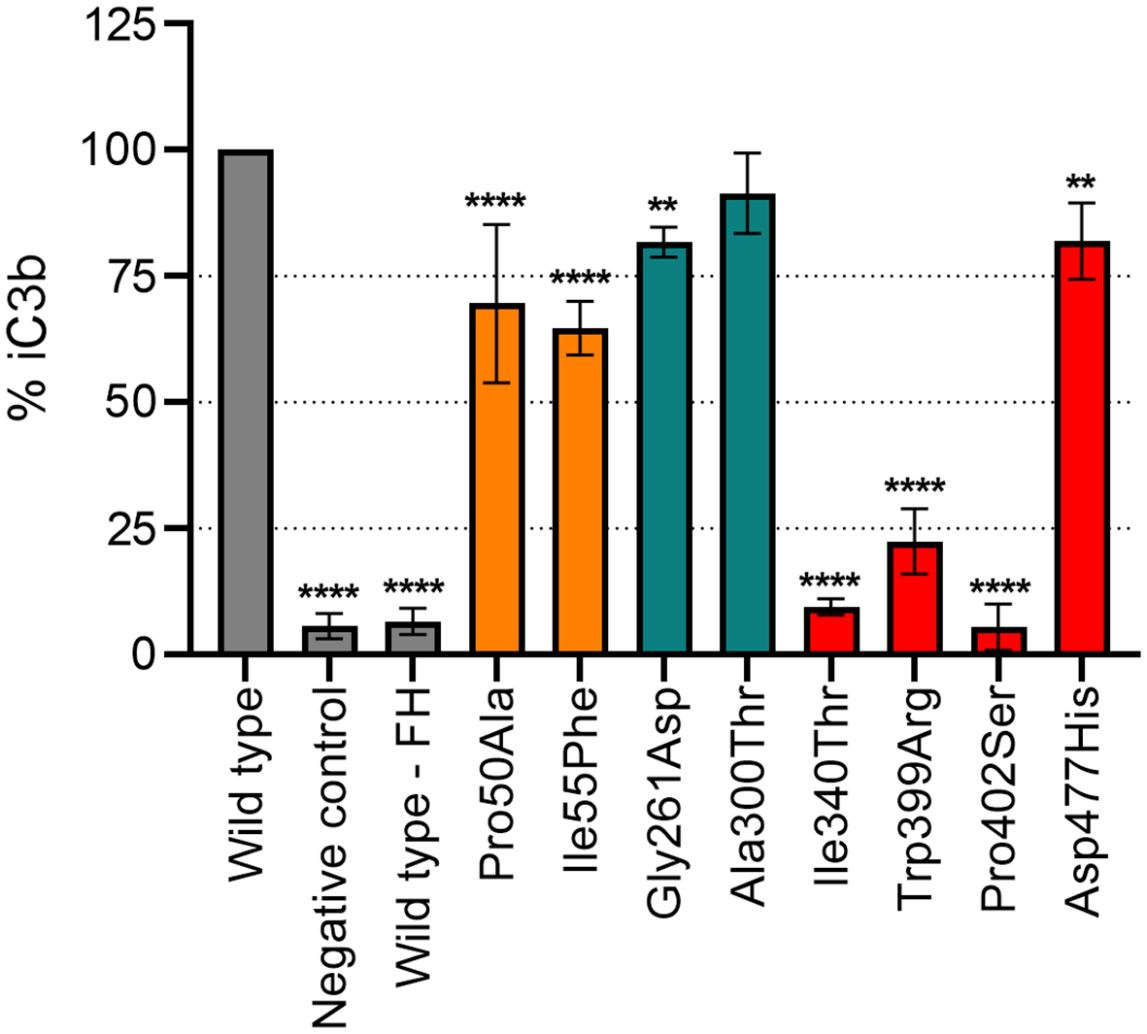
Functional analysis of recombinant FI proteins with iC3b-ELISA. Formation of iC3b was evaluated as C3b (75 µg/mL) degradation by recombinant FI variants (2 µg/mL) with FH (15 µg/mL) as a co-factor in TBS pH 7.4. The data as % iC3b is shown normalized for the recombinant wild-type (WT). Control samples are indicated in grey (WT and negative control), FI protein domains are indicated by color according to the following: FIMAC domain in orange, LDLRA2 domain in blue and SP domain in red. Samples were compared to the WT using one-way ANOVA followed by Dunnett’s multiple comparison test. The values are shown as mean +/- standard deviation of n=3. **p <0.01, and ****p <0.0001.

An ELISA specifically detecting a neoepitope in iC3b was employed to measure the percentage of iC3b generation as a result of C3b cleavage in the presence of FI with FH as co-factor (**Figure 3**). Variant Ala300Thr (LDLRA2 domain) showed similar degradation efficiency as the WT, whereas all other mutants showed significant reduction (p<0.01 or p<0.0001) in the formation of iC3b. Mutations in the SP domain displayed higher reduction in FI-activity than the ones located in the FIMAC domain; Ile340Thr, Trp399Arg and Pro402Ser resulted in the formation of approximately 80-90% less iC3b in comparison to the WT, while Pro50Ala and Ile55Phe resulted in the formation of 30% less iC3b than the WT.

### Degradation of surface-bound C3b by Luminex technology

**Figure 4:**
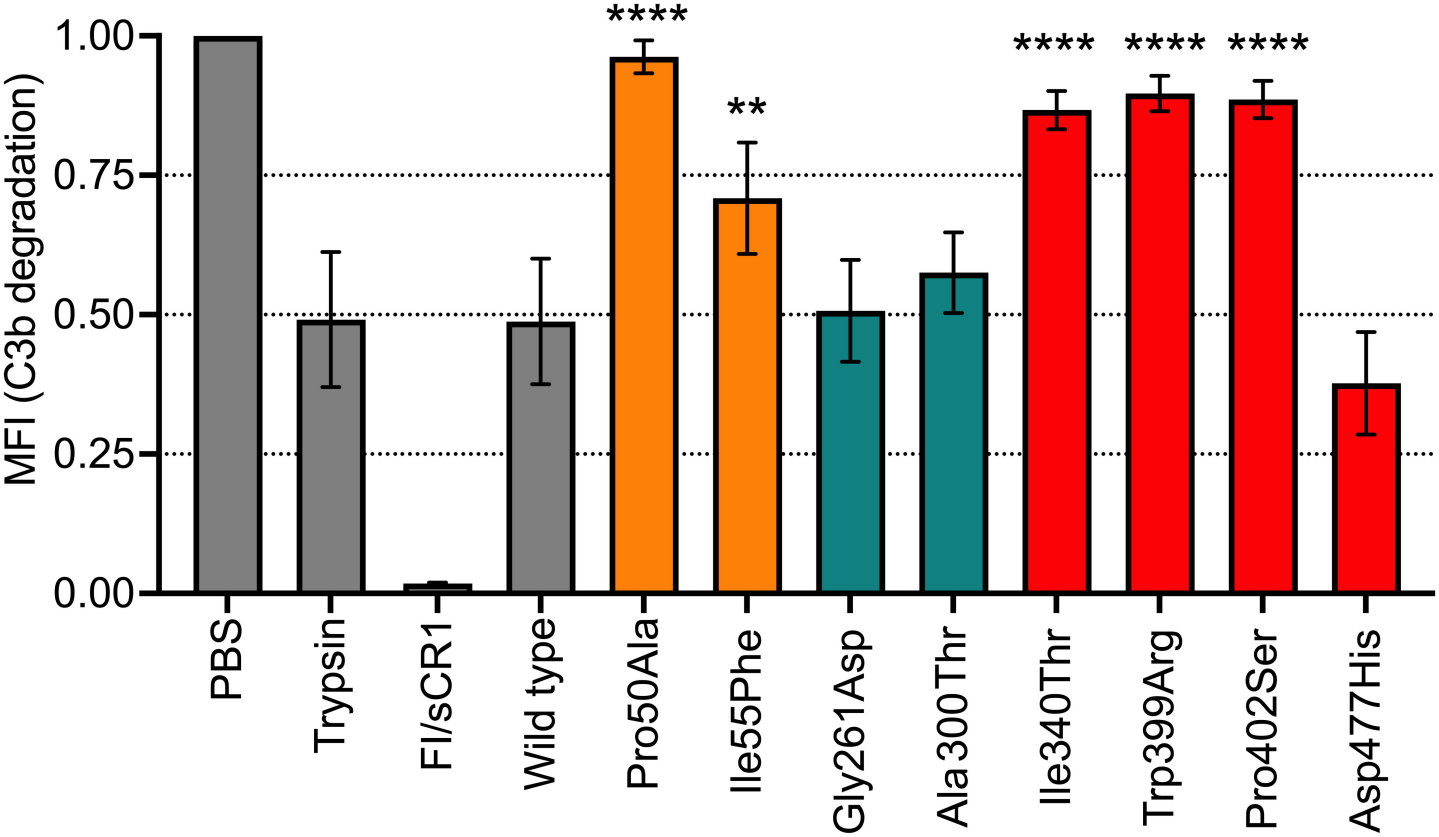
Functional analysis of recombinant FI proteins with Luminex technology. C3b was covalently bound to magnetic beads and incubated with FI recombinant proteins (0.4 μg) and sCR1 (0.4 μg) at 37°C for 60 minutes in PBS pH 7.4. C3b beads in PBS were used as the negative control sample, while C3b beads with either trypsin or purified FI and sCR1 were used as positive controls. All control samples, including wild-type (WT) control, are indicated in grey. The degradation of C3b/iC3b to C3c and C3dg was evaluated as C3c loss from the beads using a polyclonal antibody that detects C3c by measuring MFI. FI protein domains are color coded according to: FIMAC domain in orange, LDLRA2 domain in blue and SP domain in red. The values are normalized and shown as mean +/- standard deviation of n=3. **p <0.01, and ****p <0.0001.

We have established a method to study the degradation of surface-bound C3b by incubating FI with sCR1 as a co-factor and detecting loss of C3c with Luminex technology. The two FIMAC mutations that were tested; Pro50Ala and Ile55Phe showed significantly reduced degradation of C3b (p<0.0001 and p<0.01, respectively) (**Figure 4**). Furthermore, the mutations in the LDLRA2 domain (Gly261Asp and Ala300Thr) showed C3b-cleavage comparable to the WT. Three mutations in the SP domain (Il340Thr, Trp399Arg, and Pro402Ser) had significantly less degradation of C3b (p<0.0001 for all); while the Asp477His mutation showed no significant change in degradation compared to the wild type.

### Comparison of functional tests

**Figure 5:**
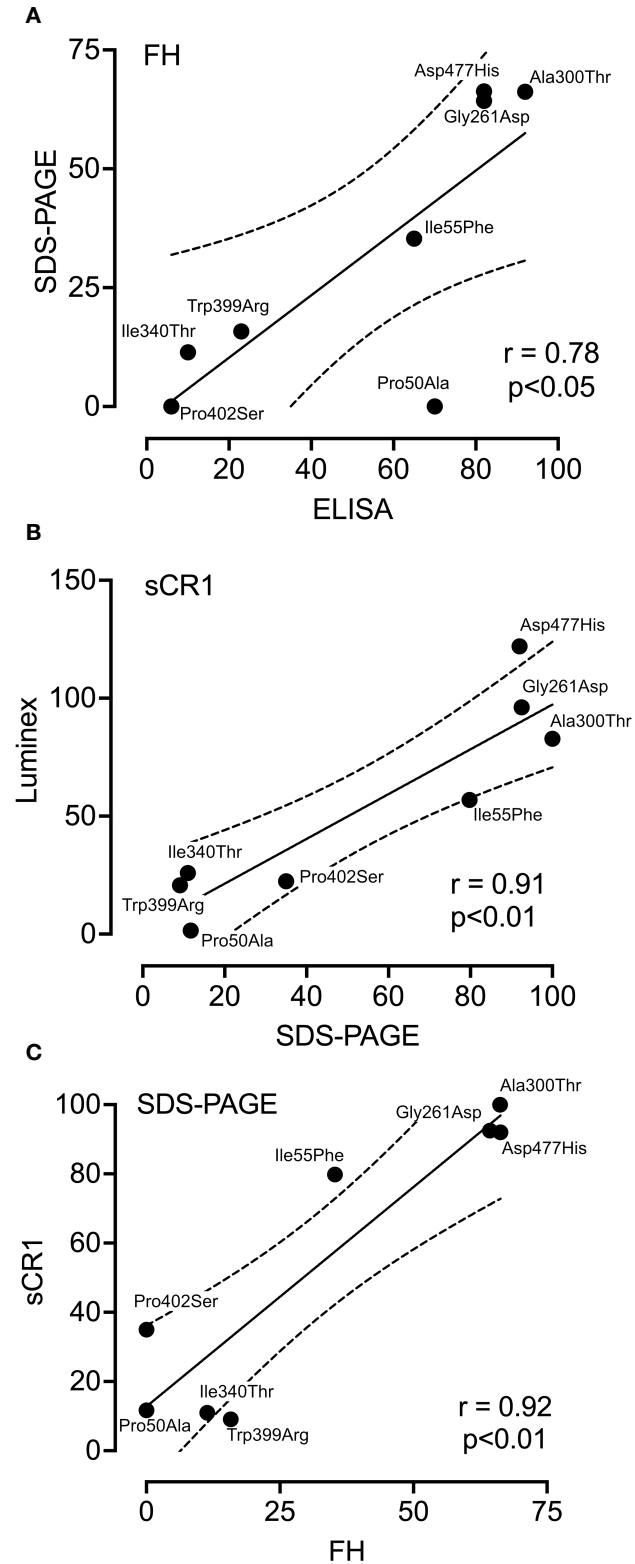
Correlation between the different co-factor activity assays. Pearson correlation analysis was performed to compare data obtained from three different functional assays; SDS-PAGE, ELISA and Luminex technology. The following pairs were analyzed; SDS-PAGE and ELISA when FH was used as a co-factor **(A)**, SDS-PAGE and luminex when sCR1 was used as a co-factor **(B)**, and SDS-PAGE in the presence of either FH or sCR1 **(C)**. r was calculated and used for the evaluation of the relationships of the variables.

To evaluate the coherence between the assays, the percentage of each variants’ activity in comparison to the wild type for each assay was summarized in **Table 2**. Subsequently, each variants’ activity was analyzed with the use of Pearson test (**Figure 5**). The iC3b ELISA and the SDS-PAGE showed a positive correlation (r=0.78, p<0.05), when using FH as a co-factor (**Figure 5A**). Similar results were found for the following variants in the LDLRA2 and SP domains; Ile340Thr, Trp399, Pro402Ser (low activity), and Gly261Asp, Ala300Thr and Asp477His (normal activity). The most profound difference was observed in regard to the FIMAC domain mutant Pro50Ala. Both assays showed significantly decreased C3b degradation for the variant, but the levels differed, where the iC3b ELISA still detected a substantial degree of iC3b formation. A comparison between the Luminex assay and the SDS-PAGE showed a strong positive correlation (r=0.91, p<0.01), when sCR1 was used as a co-factor (**Figure 5B**). Finally, the correlation between the two co-factors; FH and sCR1 in the SDS-PAGE was analyzed (**Figure 5C**). There was a strong positive correlation (r= 0.92, p=0.05). A similar degradation pattern was observed in most variants. The only variant that somewhat differed was Ile55Phe, which significantly attenuated degradation with FH, but not with sCR1.

**Table 2:** Overview of functional analysis of FI variants.

cDNA change	Protein change	SDS-PAGE		ELISA	Luminex	Classification (this study)
		FH	sCR1	FH	sCR1	
148C>G	Pro50Ala	**0** (5.1)	**12** (1.4)	**70** (15.7)	**2** (3.0)	Pathogenic
163A>T	Ile55Phe	**35** (6.2)	80 (16.1)	**65** (5.3)	**57** (10.0)	Pathogenic
782G>A	Gly261Asp	64 (8.7)	93 (9.1)	**82** (3.0)	96 (9.2)	Benign
898G>A	Ala300Thr	66 (6.6)	100 (8.5)	92 (8.0)	83 (7.2)	Benign
1019T>C	Ile340Thr	**11** (0.5)	**11** (2.8)	**10 (**1.6)	**26** (3.4)	Pathogenic
1195T>C	Trp399Arg	**16** (5.6)	**9** (5.0)	**23 (**6.4)	**21** (3.2)	Pathogenic
1204C>T	Pro402Ser	**0** (5.1)	**35** (3.8)	**6** (5.0)	**22** (3.4)	Pathogenic
1429G>C	Asp477His	66 (4.4)	92 (1.8)	**82** (7.5)	122 (9.2)	Benign

Summary of the results of the functional analysis of FI variants using three different functional assays and two cofactors (sCR1 and FH) shown in **Figures 2**–**4**. Statistically significant mean values are shown in bold type, standard deviation (+/-) is shown in brackets. Values of the ELISA, SDS-PAGE and Luminex assays are depicted in % of efficiency in comparison to the wild type FI measurement.

## Discussion

FI is a key regulator of the complement cascade and functional analysis of FI is of high clinical importance to understand disease pathophysiology related to uncontrolled complement activation, such as in AMD and aHUS (29, 30). In this study, we compared three FI functional assays to investigate the activity of eight genetic variants in FI compared to a recombinant wild-type control. We found that all mutations located in the FIMAC and the mutations within the SP domain, except Asp477His, resulted in impaired- or complete activity loss. The two mutations within the LDLRA2 domain did not impair activity. Overall, there was a large coherence between the three assays.

To elucidate whether the different FI-variants were dependent on the co-factor added for the degradation of fluid phase C3b, we performed assays using either FH or sCR1. As summarized in a recent review, recombinant FI-variants with mutations in the SP domain have been mainly associated with reduced iC3b generation and C3b co-factor activity, while mutations in the FIMAC domain tend to lead to decreased iC3b in serum. However, a great number of recombinantly expressed FI-variants has not been evaluated (31). In our experiments, the majority of the mutations occurring in the catalytic SP domain resulted in complete loss of activity of FI, with both FH and sCR1 as co-factors. The Pro50Ala mutant in the FIMAC domain, a domain important for binding to C3b (20, 32), showed significantly reduced activity in the presence of both co-factors in the fluid phase, as previously shown (12). This site is sterically close to the proposed binding site of heme, which interferes with FI’s regulatory function (27). The mutant Ile55Phe, found in both aHUS and AMD patients, resulted in impaired activity in the presence of FH, which was also confirmed by decreased iC3b levels measured with ELISA. However, with sCR1, substantial degradation of C3b was seen by the SDS-PAGE analysis, where the activity was not significantly different from the wild-type control. The SDS-PAGE analysis is based on 60-minute incubation, and with sCR1 as a co-factor, we saw a trend toward a higher C3b consumption, *i.e.*, substrate depletion and thus a lower resolution for the variants that showed some activity. Additional methods, such as real-time visualization of the formation of C3b:co-factor:FI-complex could possibly reveal whether the difference observed for Ile55Phe is a true co-factor-dependent difference (33).

To analyze whether the mutations have a different effect when FI-mediated C3b-degradation takes place on a surface in comparison to the fluid phase, we used a novel assay that allows us to quantify C3b linked to magnetic beads. In this experimental set-up, we used only sCR1 as a co-factor, as it allows degradation of iC3b to C3dg for the loss of C3c into the fluid phase. Overall, the C3b-degradation data from the Luminex assay corresponded well to what was seen by the degradation of fluid phase C3b with sCR1. The mutants in both SP and FIMAC domains, apart from Asp477His, showed strongly impaired activity. Ile340Thr and Asp477His in the SP domain were, among other genetic variants, recently evaluated in a corresponding study by de Jong et al. (12), whose data well correspond to what we found for these two variants. The mutation Ile55Phe in the FIMAC domain and Pro402Ser in the SP domain led to decreased degradation of surface-bound C3b in relation to soluble C3b. As the Ile55Phe mutation affects the contact surface with C3, this result could be explained due to reduced binding to C3b. It is reported that the enzymatic activity of FI variants is generally dependent on the binding capacity of FI to its co-factors (34). Furthermore, the Pro50Ala mutation also resulted in impaired ability of FI to degrade C3b deposited on sheep erythrocytes (34). The authors mention that substituting proline with alanine could interfere with the stability of the domain and, therefore, affect ligands that interact with surface-bound C3b.

In the last decades, the first complement inhibitors have been approved for clinical application in patients (35, 36). Understanding the functional consequence and significance of sequence variants for patient care will be essential for dedicated personalized medicine. This study provides novel information and methods concerning functional genomics for sequence variants observed in *CFI* and could assist in the interpretation of variants found in patients and improve the available tools for their diagnosis.

To summarize, we show that mutations located in the FIMAC, and SP domains impaired FI activity in various functional assays. In contrast, the two analyzed mutations in the LDLRA2 domain did not affect the function of FI to the same extent. While we demonstrated that the mutations affected the activity of FI in a similar manner independently of most of our experimental set-ups, some differences could only be detected by employing different methods or co-factors. For instance, attenuated activity for the Gly261Asp could only be detected in the iC3b ELISA, which shows the value of combining methods in the diagnostic testing. However, further functional studies with a larger number of variants in different domains of *CFI* are needed to further investigate the effect of the use of different cofactors on C3b degradation activity, and the impact on degradation of surface bound and fluid phase C3b by FI.

## Data availability statement

The original contributions presented in the study are included in the article/**Supplementary Material**. Further inquiries can be directed to the corresponding author.

## Ethics statement

Ethical approval was not required for the studies on humans in accordance with the local legislation and institutional requirements because only commercially available established cell lines were used.

## Author contributions

LB: Writing – original draft, Investigation, Methodology. AG: Investigation, Methodology, Writing – original draft. DS: Writing – review & editing, Methodology. NK: Writing – review & editing, Funding acquisition, Resources. MP: Investigation, Writing – review & editing, Methodology. SP: Investigation, Writing – review & editing. PN: Writing – review & editing, Conceptualization, Project administration, Supervision, Resources. LH: Project administration, Supervision, Writing – review & editing, Conceptualization, Resources.
