# Supplementary material for: Functional evaluation of complement factor I variants by immunoassays and SDS-PAGE
Source: Front Immunol. 2023 Oct 26;14:1279612. doi: 10.3389/fimmu.2023.1279612 (PMC10639126; doi:10.3389/fimmu.2023.1279612)
Supplement: Supplementary file 1 [file DataSheet_1.docx]

Supplementary Material

Functional evaluation of complement factor I variants by immunoassays and SDS-PAGE

**Alexandra Gerogianni†, Laura M. Baas†, Dick J. Sjöström, Nicole C.A.J van de Kar, Marit Pullen, Siem J. van de Peppel, Per H. Nilsson‡, Lambertus P. van den Heuvel‡*.**

***Correspondence: Lambertus P. van den Heuvel, bert.vandenheuvel@radboudumc.nl**

# Supplementary Figures and Tables

## Supplementary Figures


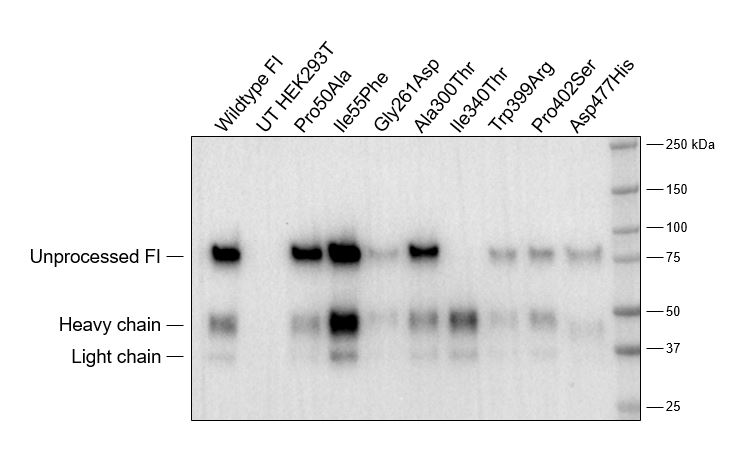


**Supplementary Figure 1. Western blot analysis of recombinant proteins.** Proteins were run on SDS-PAGE under reducing conditions and presence of unprocessed FI (88kDa), the FI heavy chain (50 kDa) and the FI light chain (38 kDa) were confirmed with a polyclonal goat anti-human FI antibody. Abbreviations used: FI; Factor I, UT; Untreated

## Supplementary Tables

**Supplementary Table 1. Primer sequences used for point mutagenesis.** Primer sequences used for point mutagenesis. Protein numbering includes the signal peptide. The base altered by site-directed mutagenesis is indicated in red. For the reverse primer, either overlapping primers (two altered bases/primer set; indicated by ^*^) or non-overlapping primers (one altered base/primer set; indicated by ^**^) was used.
